# Gene flow and Andean uplift shape the diversification of *Gasteracantha cancriformis* (Araneae: Araneidae) in Northern South America

**DOI:** 10.1002/ece3.4237

**Published:** 2018-06-25

**Authors:** Fabian C. Salgado‐Roa, Carolina Pardo‐Diaz, Eloisa Lasso, Carlos F. Arias, Vera Nisaka Solferini, Camilo Salazar

**Affiliations:** ^1^ Programa de Biología Facultad de Ciencias Naturales y Matemáticas Universidad del Rosario Bogotá Colombia; ^2^ Departamento de Ciencias Biológicas Universidad de los Andes Bogotá Colombia; ^3^ Smithsonian Tropical Research Institute Ancón Panamá; ^4^ Department of Genetics, Evolution and Bioagents Institute of Biology University of Campinas Campinas Sao Paulo Brazil

**Keywords:** Andean passes, Andean uplift, Colombian Andes, *Gasteracantha cancriformis*, gene flow

## Abstract

The Andean uplift has played a major role in shaping the current Neotropical biodiversity. However, in arthropods other than butterflies, little is known about how this geographic barrier has impacted species historical diversification. Here, we examined the phylogeography of the widespread color polymorphic spider *Gasteracantha cancriformis* to evaluate the effect of the northern Andean uplift on its divergence and assess whether its diversification occurred in the presence of gene flow. We inferred phylogenetic relationships and divergence times in *G. cancriformis* using mitochondrial and nuclear data from 105 individuals in northern South America. Genetic diversity, divergence, and population structure were quantified. We also compared multiple demographic scenarios for this species using a model‐based approach (phrapl) to determine divergence with or without gene flow. At last, we evaluated the association between genetic variation and color polymorphism. Both nuclear and mitochondrial data supported two well‐differentiated clades, which correspond to populations occurring on opposite sides of the Eastern cordillera of the Colombian Andes. The final uplift of this cordillera was identified as the most likely force that shaped the diversification of *G. cancriformis* in northern South America, resulting in a *cis*‐ and *trans*‐Andean phylogeographic structure for the species. We also found shared genetic variation between the *cis*‐ and *trans*‐Andean clades, which is better explained by a scenario of historical divergence in the face of gene flow. This has been likely facilitated by the presence of low‐elevation passes across the Eastern Colombian cordillera. Our work constitutes the first example in which the Andean uplift coupled with gene flow influenced the evolutionary history of an arachnid lineage.

## 1 INTRODUCTION

The northern Andes in South America is one of the most biodiverse regions on the planet, and the origins of this rich diversity have been linked to past geological and climatic events such as the uplift of the Andes and quaternary climatic oscillations (Kattan, Franco, & Rojas, 2004). The effect of these geoclimatic events in promoting divergence between Neotropical populations and species can be elucidated using genetic data, especially by detecting deviations from the expected coalescent patterns in neutral loci (Rull, 2011). Most studies addressing this question have identified the uplift of the northern Andes as a major driver of Neotropical diversification in a scenario consistent with allopatric differentiation, wherein the complex topography of the Andes isolated populations on both sides of this barrier thus restricting gene flow (Antonelli et al., 2018; Brumfield & Capparella, 1996; Hoorn et al., 2010). In contrast with this predominant view, a recent comparative phylogeographic study found discordant divergence times for multiple avian lineages with cross‐Andean distribution, a result that is better explained by dispersal ability across the Andes rather than a single vicariant event (Smith, Harvey, Faircloth, Glenn, & Brumfield, 2014; Smith, McCormack et al., 2014). In line with this finding, new evidence supports the notion that common diversification modes in Neotropical birds include secondary contact between cross‐Andean populations or between divergence in the presence of gene flow, facilitated by low‐elevation corridors along the Andes (Cadena, Pedraza, & Brumfield, 2016; Oswald, Overcast, Mauck, Andersen, & Smith, 2017).

However, our current knowledge on the modes of animal diversification in the northern Andes is mostly based on vertebrates, and despite arthropods being the most diverse group of animals, analyses of their diversification in this region remain scarce (De‐silva et al., 2017; Turchetto‐Zolet, Pinheiro, Salgueiro, & Palma‐Silva, 2013). Some studies limited to insects, especially butterflies, show that the Andean mountains played an important role in their diversification, where speciation with and without gene flow across the Andes has occurred (Arias et al., 2012; Chazot et al., 2016, 2017; De‐silva et al., 2017; Díaz et al., 2014; Dick, Roubik, Gruber, & Bermingham, 2004; Elias et al., 2009). Despite this, a comprehensive understanding on how the Andean orogeny has promoted Neotropical animal diversification requires the inclusion of additional arthropod taxa, such as arachnids.

*Gasteracantha cancriformis* (Linnaeus, 1758) is a common Neotropical orb‐web spider that exhibits sexual dimorphism, with females being up to four times larger than males (5 to 13 mm vs 2 to 3 mm, respectively; Muma, 1971). Furthermore, while males apparently spin no definite web, females build a stronger net with reinforced guylines, distinct tufts of silk on the foundation lines and a definitive central disk (Muma, 1971). This orb‐web varies in size and position, and is used to capture prey such as whiteflies, flies, moths, and beetles (Muma, 1971). The species is also characterized by its color polymorphism, with at least eight known morphs (Gawryszewski, 2007), but the causes maintaining this variation remain unknown (Gawryszewski & Motta, 2012).

*Gasteracantha cancriformis* has a wide distribution in the Neotropical and subtropical region (from the south of the United States to northern Argentina; Levi, 1978) and consequently occurs on both sides of the Andes and in the Colombian inter‐Andean valleys. This distribution makes it an excellent model to test whether the uplift of the Andes has influenced its diversification at the population level. For example, the Andean uplift may have fragmented populations at opposite sides, thus limiting genetic connectivity; this scenario predicts reciprocally monophyletic clades at each flank of the Andes. Otherwise, long‐distance migration of individuals across dispersal corridors in the Andes could favor gene flow among areas; this scenario of divergence with gene flow predicts monophyletic groups at opposite sides of the Andes sharing genetic variation between them. At last, rampant migration of individuals across the Andes could promote genetic homogenization of populations in spite of the geographic barrier; in this scenario, it is expected to find population clustering not explained by geography.

**Figure 1:**
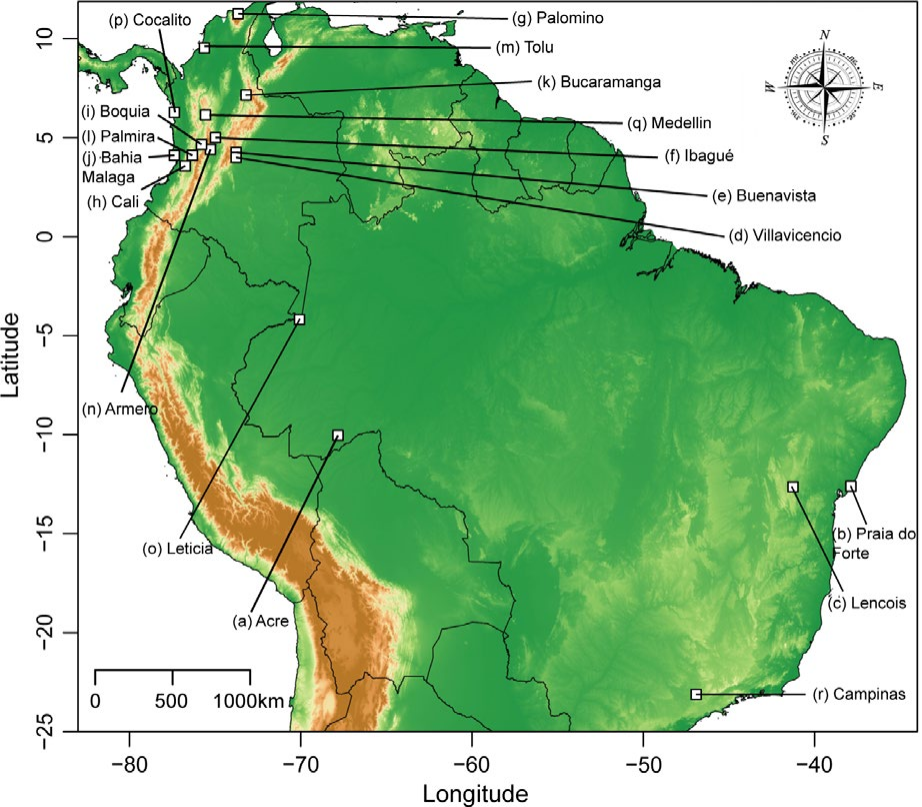
Map of the Neotropics showing the 18 sampling localities in Colombia and Brazil. White squares correspond to each locality sampled. Sampled localities are (a) Acre, (b) Praia do forte, (c) Lencois, (d) Villavicencio, (e) Buenavista, (f) Ibague, (g) Palomino, (h) Cali, (i) Boquia, (j) Bahia Malaga, (k) Bucaramanga, (l) Palmira, (m) Tolu, (n) Armero, (o) Leticia, (p) Cocalito, (q) Medellin, and (r) Campinas

Here, we implemented a multilocus approach to study the genetic connectivity between polymorphic populations of *G. cancriformis* across the northern Andes (Figure 1, Supporting information Table S1) and tested scenarios of strict vicariance versus divergence with gene flow. We also evaluated whether lineage clustering in this spider is explained by geography or color pattern. Overall, this work contributes to deepen our understanding of how Andean orogeny has shaped processes of biotic diversification and biogeography in the Neotropics.

## 2 MATERIALS AND METHODS

### 2.1 Sample collection

We used standard aerial searching and beating methods to sample a total of 105 individuals of *G. cancriformis* from 17 localities distributed from the north of Colombia to southeastern Brazil (Figure 1, Supporting information Table S1). Specimens were color coded following the categorization established by Gawryszewski (2007), preserved in a 20% dimethylsulfoxide (DMSO) solution saturated with NaCl, and stored at −80°C. Colombian samples were deposited in the “Colección de Artrópodos de la Universidad del Rosario” (CAUR#229), and Brazilian specimens were deposited in the “Coleção Científica de Aracnídeos e Miriápodes of the Instituto Butantan” (São Paulo, Brazil).

### 2.2 DNA extraction, amplification, and sequencing

DNA was extracted from legs using the Wizard Genomic DNA purification kit (Promega), following the manufacturer's protocol. We amplified two mitochondrial genes, the cytochrome oxidase I (587 pb; COI; Folmer, Black, Hoeh, Lutz, & Vrijenhoek, 1994) and the 16S ribosomal DNA gene (397 pb; 16s; Simon et al., 1994), using the conditions reported in previous studies (Blackledge et al., 2009; McHugh, Yablonsky, Binford, & Agnarsson, 2014; Peres et al., 2015). We also sequenced two nuclear loci, the internal transcribed spacer 2 (296 pb; ITS; White, Bruns, Lee, & Taylor, 1990) and 28S ribosomal DNA gene (681 pb; 28s; Hedin & Maddison, 2001), using primers previously developed for spiders (Moradmand, Schönhofer, & Jäger, 2014; Peres et al., 2015). At last, we used the published transcriptome of *G. cancriformis* (Prosdocimi et al., 2011) and designed the forward primer (5′‐ CAATTACACCTGGGAATCTTCTG‐3′) and reverse primer (5′‐CCCTGACAAAATTCAAATAGTCG‐3′) to amplify a 560‐bp fragment of the heat shock protein 90 (HSP90), a gene that was used previously in phylogenetic studies in *Heliconius* (Salazar et al., 2010). PCR reactions for this marker were performed in a 10 μl reaction volume containing 1× PCR buffer, 2.5 mM MgCl_2_, 500 μM dNTPs, 0.5 μM each primer, 0.5 U Platinum Taq (INVITROGEN), and 30–40 ng of DNA. The PCR cycling profile was 94°C for 5 min, 40 cycles at 94°C for 30 s, Tm 54°C for 30 s, and 72°C for 1 min and final extension at 72°C for 10 min. For all loci, we visualized 1 μL of the PCR reaction in a 1.5% agarose gel to verify the success of PCR. Positive amplicons were cleaned by ExoSAP‐IT (USB Corp., Cleveland, OH), and their sequencing was carried out by ELIM Biopharmaceuticals Inc. (Hayward, CA).

Gene sequences were read, edited, and assembled with CLC main workbench (QIAGEN) to obtain a consensus sequence per individual. Haplotype inference for heterozygous calls was conducted for nuclear loci with the PHASE algorithm implemented in dnaSP v5.10 (Librado & Rozas, 2009) with 5000 iterations per simulation and accepting inferred haplotypes with a confidence higher than 90%. Then, we used mega 6.0 (Tamura, Stecher, Peterson, Filipski, & Kumar, 2013) using the clustal W algorithm (Thompson, Higgins, & Gibson, 1994) to create alignments for each locus; these alignments were visually inspected and corrected accordingly. Alignments were translated to protein and verified for stop codons using mesquite v3.04 (Maddison & Maddison, 2015).

### 2.3 Molecular phylogenetics and divergence times

The nucleotide substitution model for each mitochondrial gene was selected using the BIC criterion in jmodeltest 0.1.1 (Posada, 2008). The most suitable models were HKY+I for COI and TIM+I for 16S.

Tree topologies were estimated with Bayesian inference (BI) using beast 1.7.4 (Drummond, Suchard, Xie, & Rambaut, 2012) and including two *Micrathena vigorsi* individuals as outgroups (Supporting information Table S1). We unlinked the substitution model for each gene and linked the clock model and the tree model. We applied a lognormal relaxed clock to estimate divergence times using a mutation rate of 0.0112 (*SD* = 0.001) substitution/site/million years previously reported for node dating and calibration in spiders (Bartoleti et al., 2017; Bidegaray‐Batista & Arnedo, 2011; Kuntner, Arnedo, Trontelj, Lokovšek, & Agnarsson, 2013). We ran two runs of 100 million generations sampling every 1000 generations. The initial 10,000 trees were discarded as burn‐in using treeannotator (Drummond & Bouckaert, 2015). We examined the output in tracer 1.7 (Rambaut, Drummond, Xie, Baele & Suchard, 2018) to confirm that all effective sample size (ESS) values were >200 and to confirm the convergence of the chains to the stationary distribution. The maximum credibility tree that best represented the posterior distribution was visualized and edited with figtree 1.4 (Rambaut, 2012).

Phylogenetic reconstruction was also performed with maximum likelihood (ML) in IQ‐tree (Nguyen, Schmidt, Von Haeseler, & Minh, 2015) using the same substitution models described before and applying the edge‐proportional partition. Node support was assessed with 1000 bootstrap replicates. We also constructed haplotype median‐joining networks per locus with popart (Leigh, Bryant, & Nakagawa, 2015).

### 2.4 Population genetics

We calculated haplotype diversity (h), nuclear diversity (π), number of segregating sites (ss), and Tajima's D with dnaSP v5.10 (Librado & Rozas, 2009). Genetic structure was evaluated using *F*_ST_ at two different levels: among phylogenetic clades (i.e., populations occurring in opposite sides of the Eastern cordillera of the Colombian Andes) and among populations. Significance of deviations from panmixia was assessed with the Hudson permutation test (Hudson, Boos, & Kaplan, 1992). An analysis of molecular variance (AMOVA) was also calculated for the same levels of differentiation with arlequin 3.5 (Excoffier & Lischer, 2010) using 10,000 permutations.

Using the nuclear dataset, we identified the number of population genetic clusters (K) with the Bayesian clustering approach implemented in structure 2.3.4 (Pritchard, Stephens, & Donnelly, 2000). We ran the analysis under the admixture model with a 50,000 burn‐in, 100,000 MCMC sampling generations for K ranging from 1 to 13 (localities with only one individual were removed from this analysis), and 20 iterations for each value of K. We determined the best K by applying three complementary approaches as recommended by Janes et al. (2017): (i) according to the Δ K method of Evanno (Evanno, Regnaut, & Goudet, 2005), (ii) plotting the likelihood of K for each value of K (Earl & vonHoldt, 2012), and (iii) reporting multiple barplots for K values between 2 and 5. All these tests were implemented in clumpak (Kopelman, Mayzel, Jakobsson, Rosenberg, & Mayrose, 2015). We ran a multivariate analysis in order to make an additional validation of the genetic clusters for each locus. We did this by transforming fasta sequences into a genind object and loading it into the *adegenet* R package (Jombart & Ahmed, 2011), in which we performed a principal component analysis (PCA). We retained the first two components for a subsequent canonical discriminant analysis using the R package *candisc* (Friendly & Fox, 2017).

As isolation by distance (IBD) can obscure population structure signals, we investigated the presence of IBD for each locus using the Mantel (Mantel, 1967) with the R package *vegan* (Dixon, 2003). In order to do this, pairwise geographic distances among localities were calculated with the function *distm* from the R package *geosphere* (Hijmans, 2016), while genetic distances were estimated by linearizing the *F*_ST_ values previously obtained. We also implemented a partial Mantel test (Smouse, Long, & Sokal, 1986) to separate the effect of geographic distance from the population assignments based on structure results.

Considering the recently highlighted limitations of the Mantel test (Legendre & Fortin, 2010; Legendre, Fortin, & Borcard, 2015; Meirmans, 2012), we also tested linear correlations between the logarithm of the geographic distances and genetic distances as recommended by Legendre and Fortin (2010) and Diniz‐filho et al. (2013).

### 2.5 Demographic model testing and parameter estimation

We used phrapl (Jackson, Morales, Carstens, & O'Meara, 2017) to choose a demographic model that fits our data. phrapl compares the topologies obtained from empirical data with those simulated under multiple demographic models and then, by calculating the proportion of times that simulated topologies match the empirical ones, it approximates the log‐likelihood of the data under a given model. phrapl calculates Akaike information criterion (AIC) as a measure of lack of model fit while the associated AIC weights (wAIC) can be interpreted as model probabilities.

phrapl can compare various demographic models, which can be broadly categorized as (a) isolation only (IO), (b) migration only (MO), (c) isolation with migration (IM), and (d) mixed models (ME). We tested six models that fall into the IO and IM categories. The first is an IO model with a single coalescent event and no migration, while the other five are IM models that assume constant gene flow along the branch length and differ in the direction and strength of migration.

As input for phrapl, we assigned each individual to its collection site (east or west of the Eastern cordillera) and loaded five midpoint rooted trees estimated with IQ‐tree (one per locus; Nguyen et al., 2015). We used jmodeltest (Posada, 2008) to select the most plausible pattern of sequence evolution for each gene (COI: HKY+I, 16S: TIM+I, ITS: K80 + I, 28S: F81 + I, HSP90: GTR+I).

We subsampled four tips per group and 100 subsamples per gene, giving a total of 500 observed trees. Then, following previous studies (Denton, Morales, & Gibbs, 2018; Hime et al., 2016; Morales, Jackson, Dewey, Meara, & Bryan, 2017), we conducted a simulation of 100,000 gene trees using a grid of parameter values for divergence time (*t* = 0.30, 0.58, 1.40, 2.54, 4.1) and migration (*m* = 0.10, 0.22, 0.46, 1.00, 2.15), in units of 4 N and 4 Nm, respectively. This array was designed to encompass the full range of potential values in each dataset as such information is not available yet for *G. cancriformis*. When we detected gene flow in our dataset, we tested two additional models that correspond to recent (τ‐9τ/10) and ancient secondary contact (τ‐τ/5), starting from the tips.

Using the model selected by phrapl, we estimated population sizes (θ), divergence time (τ), and migration rates (*m*) with the Bayesian coalescent program G‐phoCS (Gronau, Hubisz, Gulko, Danko, & Siepel, 2011). We ran independent analyses for mtDNA and nDNA, each with 50,000 burn‐in iterations and 500,000 additional sampling iterations; runs showed adequate mixing and convergence under these conditions. As G‐phoCS uses gamma distributions (α, β) to specify the prior distributions for the parameters, we tested deep (α = 1 and β = 30) and shallow (α = 1 and β = 300) divergence, both with migration rate priors set to α = 1 and β = 10. As results for both were very similar, we are presenting only those of β = 30. For the mitochondrial analysis, we converted raw parameter estimates using the mitochondrial rate of 0.0112 substitutions/site/million years and the nuclear rate of 0.0084 substitutions/site/million years (Bidegaray‐Batista & Arnedo, 2011).

### 2.6 Phenotype by genotype association

To test whether there is an association between the coloration of individuals and their genetic variation, we ran a chi‐square Monte Carlo test under the null hypothesis of independence between coloration and genetic haplotypes. This analysis was run for each locus and establishing color morphs categories following the categories of Gawryszewski (2007). The analysis included only Colombian specimens, due to the fact that Brazilian samples do not have color records.

## 3 RESULTS

### 3.1 Phylogenetic relationships and divergence time

**Figure 2:**
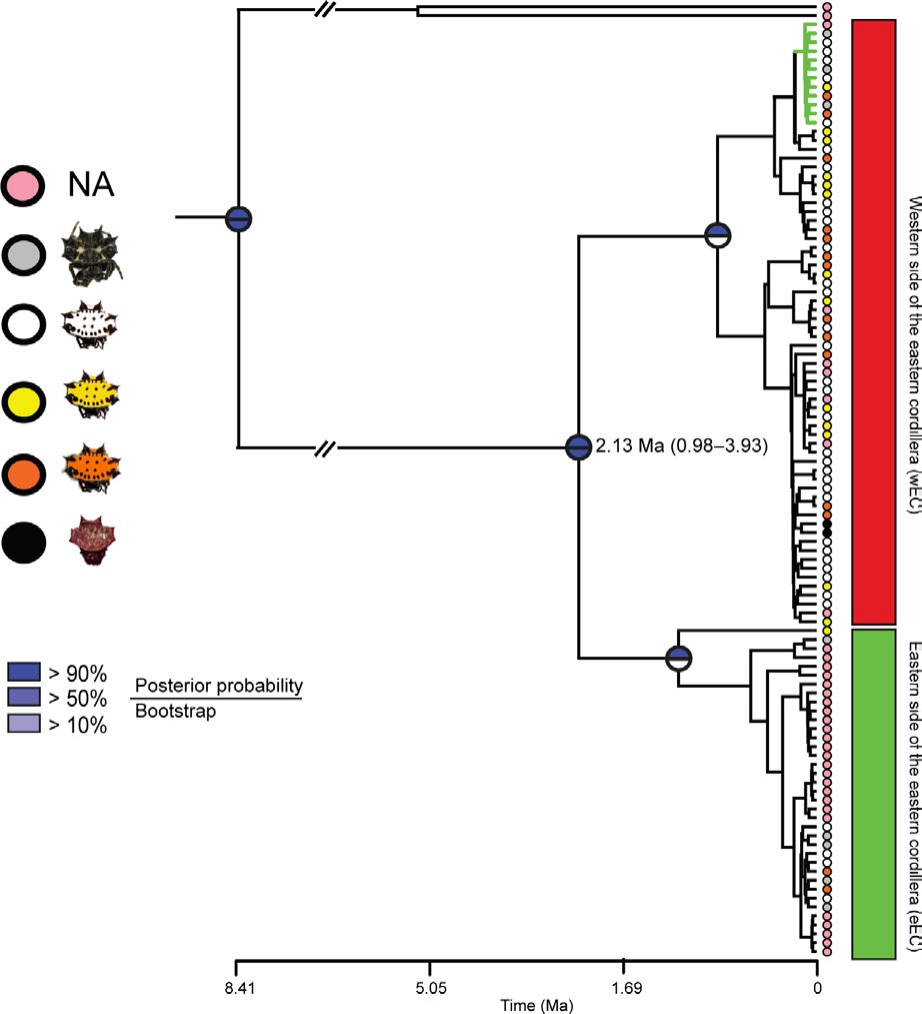
Mitochondrial phylogeny. Best recovered tree with mtDNA where node supports are represented by circles divided into two: The upper half corresponds to posterior probabilities obtained by Bayesian inference, and the lower half to the maximum‐likelihood bootstrap values after 1,000 bootstrap pseudoreplicates. Colored circles at the tips represent the color phenotype in the opisthosoma of each individual. Green and red squares highlight the eastern (eEC) and western (wEC) sides of the Eastern Cordillera of the Colombian Andes. Green branches highlight individuals sampled in the eEC that cluster into the wEC clade

Both BI and ML showed the same phylogenetic pattern which revealed two highly supported clades, but with their internal relationships unresolved (Figure 2). These major clades correspond to populations at the eastern (eEC) and western (wEC) sides of the Eastern Cordillera of the Colombian Andes (EC), with some individuals from the foothills of the EC showing shared haplotypes between both clades (Figure 2). Other physiographic features of the Neotropics, such as the Western and Central Cordilleras of the Colombian Andes or the Brazilian Dry Diagonal, do not cause population structure in *G. cancriformis*. Therefore, Brazilian samples were monophyletic within the eEC clade (Figure 2). beast estimated a mitochondrial divergence time for the two main clades at *ca*. 2.13 Ma (95% HPD = 0.98–3.93 Ma; Supporting information Figure S1). This estimate largely coincides to that of G‐phoCS using mitochondrial data, where divergence time was 1.68 Ma (95% HPD = 0.66–2.53 Ma; Supporting information Figure S1 and Table S2). Both of these dates are very close to the Pliocene/Pleistocene boundary and concordant with the final EC uplift (Gregory‐Wodzicki, 2000). When using nuclear data, G‐phoCS estimated a divergence time of 0.66 Ma (95% HPD = 0.37–0.96 Ma; Supporting information Figure S1).

### 3.2 Population genetics

Mitochondrial and nuclear median‐joining networks revealed two groups with shared haplotypes, corresponding to the eEC and wEC clades (Supporting information Figure S2). These clades are separated by 25 mutational steps in the mitochondrial marker COI and by 10 mutational steps in the mitochondrial marker 16S. In nuclear loci, the groups are differentiated by one mutation (Supporting information Figure S2). These groups are genetically differentiated as shown by our *F*_ST_ analysis (*F*_ST__mtDNA = 0.60; *F*_ST__ITS = 0.24; *F*_ST__28S = 0.25; *F*_ST__HSP90 = 0.20; for all loci *p* < 0.05 in the Hudson permutation test).

Mitochondrial nucleotide diversity was higher in the eEC clade than in the wEC clade (Table 1); however, this did not hold true for nuclear loci, which may be due to differences in effective size and other causes of mito‐nuclear discordance (Toews & Brelsford, 2012). None of the loci showed significant Tajimas’ D suggesting neutral evolution (Table 1). For all loci, genetic structure was more pronounced among populations sampled at different sides of the EC than among populations at the same side (Supporting information Figure S3). This pattern was also reflected in the AMOVA analysis, where most of the mitochondrial variation is explained by differences among regions (eEC and wEC clades; Supporting information Table S3). However, for nuclear loci, most of the variance is due to differences within populations.

**Table 1:** Population genetic summary statistics for the eastern (eEC) and western (wEC) sides of the Eastern Colombian cordillera for each locus

Pop	Mitochondrial					ITS					28S					HSP90				
	*N*	*S*	π	*H*_d_	*D*	*N*	*S*	π	*H*_d_	*D*	*N*	*S*	π	*H*_d_	*D*	*N*	*S*	π	*H*_d_	*D*
eEC	49	67	0.022	0.896	1.648	46	12	0.008	0.835	−0.021	49	7	0.002	0.703	−0.342	40	10	0.002	0.729	−1.435
wEC	56	45	0.007	0.927	−0.936	53	11	0.007	0.871	−0.606	57	10	0.002	0.72	−0.459	52	23	0.005	0.855	−1.078

*Notes*

Pop: Population; *N*: number of sequences; *S*: number of polymorphic sites; π: nucleotide diversity; *H*_d_: haplotype diversity; *D*: Tajima's D.

None of the loci showed Tajima's D values that departed from neutral expectations.

**Figure 3:**
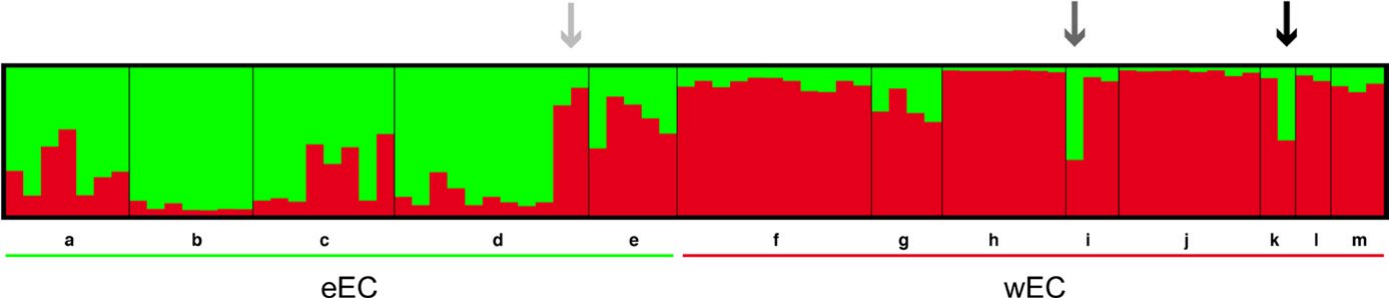
Bayesian population assignment test based on nDNA. A population assignment test with the software structure based on three nuclear loci identified two distinct populations (K = 2). Bar plots show Bayesian assignment probabilities for individuals where each color represents the most likely ancestry from which the genotype was derived (green: eEC and red: wEC). Bars on the bottom indicate the geographic region that each population belongs to. Populations are coded as described in Figure 1. In population d, individuals 67 and 68 (light gray arrow) have almost 80% of their nDNA from wEC. Individual 78 in population i (mid gray arrow) and individual 95 in population m (black arrow) have wEC mtDNA, but their nDNA showed almost 50% of shared ancestry with the eEC populations

All methods applied to select the optimal value of K consistently revealed two groups (K = 2; Figure 3, Supporting information Figures S4 and S5), which is consistent with the phylogenetic analyses and the population pairwise *F*_ST_ values (Figure 2, Supporting information Figure S3). In agreement with those results, the canonical discriminant analyses also identified two geographic clusters (Supporting information Figure S6). Individuals from both groups share variation between them, for example, most individuals from Villavicencio (eastern foothills of the EC) had either wEC or eEC mtDNA, and up to 30% of their nDNA was of wEC origin. Furthermore, there were two individuals from this locality with wEC mtDNA, and almost ~80% of their nDNA was of wEC origin (Figures 2 and 3, Supporting information Figure S7). Likewise, two individuals from Boquia and Bucaramanga (West and Central Cordillera, respectively) presented wEC mtDNA, but their nDNA showed a shared ancestry of almost 50% with the eEC populations (Figures 2 and 3; Supporting information Figure S7). We ruled out any effect of isolation by distance rather than Andean divergence causing the geographic population structure observed here (Supporting information Figure S8 and Table S4).

### 3.3 Demographic models

**Figure 4:**
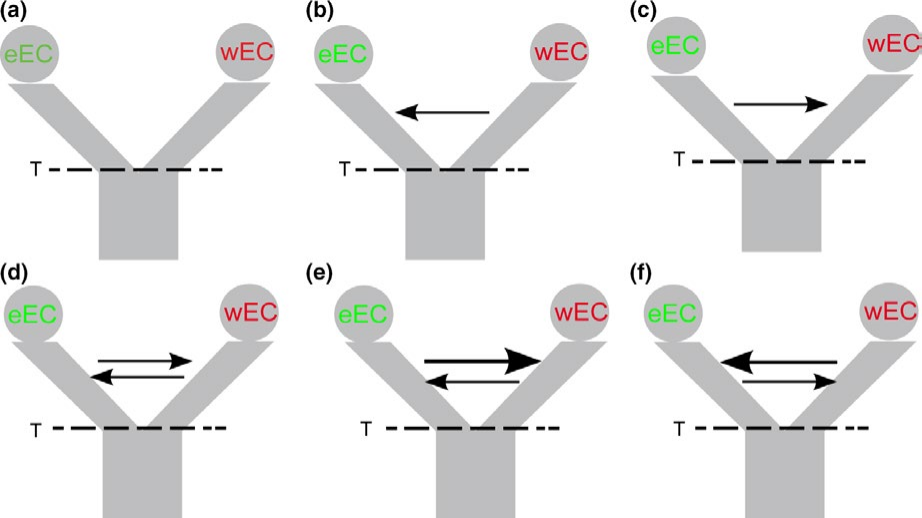
Demographic scenarios tested for the evolution of *G. cancriformis* in the northern Andes. (a) Divergence with no migration, (b) divergence with unidirectional migration from wEC to eEC, (c) divergence with unidirectional migration from eEC to wEC, (d) divergence with bidirectional symmetrical migration, (e) divergence with bidirectional asymmetrical migration from eEC to wEC, and (f) divergence with bidirectional asymmetrical migration from wEC to eEC

phrapl revealed different wAIC values and high ∆AIC between models with gene flow (IM) and isolation only (IO; Table 2 and Figure 4; Supporting information Table S5). The model with no migration had the lowest wAIC indicating that a single vicariant event with no genetic exchange is not plausible. However, it is difficult to differentiate between symmetrical and asymmetrical gene flow (Table 2; Supporting information Table S5). When we tested recent versus ancient secondary contact, the latter model was better supported, ruling out recent secondary contact but suggesting at least some isolation caused by the vicariant event (Table 2). These results imply that gene flow is responsible for the shared ancestry of *G. cancriformis* between eEC and wEC geographic regions. Likewise, G‐phoCS also detected reciprocal gene flow between individuals in these two regions (Supporting information Table S2).

**Table 2:** Measures of fit of alternative models, labeled as in Figure 4

Model	AIC	lnL	*K*	∆AIC	wAIC
a	114.20	−56.10	1	15.66	1.50E−04
b	101.65	−48.82	2	3.10	0.08
c	102.26	−49.13	2	3.72	0.06
d	99.70	−47.85	2	1.16	0.21
e	101.72	−47.86	3	3.18	0.08
f	101.78	−47.89	3	3.24	0.07
g	100.88	−47.85	2	2.34	0.18
h	98.54	−47.27	2	0.00	0.38

*Note*

Isolation only (a), isolation with migration (b to f), and secondary contact (g and h).

### 3.4 Phenotype by genotype association

We found the white phenotype to be the most frequent morph among all populations studied, while the black–white morph was only present in the eEC populations. Nonetheless, in the Colombian Cauca valley (wEC), we collected a black morph that had not been previously reported (Supporting information Figure S9). Although our molecular sampling (mtDNA and nDNA) revealed a statistical association between genetic variation and geography, such association was not found for color polymorphism (Supporting information Table S6). This lack of association is also evident in the mtDNA phylogeny, as individuals of different colors cluster within same clade (Figure 2).

## 4 DISCUSSION

Our mitochondrial and nuclear data showed two well‐supported genetic clusters separated by the EC of the Colombian Andes, although this pattern was more evident in mtDNA than in nDNA. This discordance is likely due to the differences in effective population sizes between nDNA and mtDNA, the latter having four times less effective population size causing it to complete the process of lineage sorting faster and, as a consequence, estimate older coalescent times (Toews & Brelsford, 2012). In contrast, the larger effective population size of nDNA causes not only younger coalescent times but also the co‐existence of multiple haplotypes with minor differences among them, which increases the “within population” variance, as reflected in our AMOVA analysis. This mito‐nuclear discordance is not exclusive to our case but has also been observed in other studies which found the Andes mountains to play a role in splitting populations (Smith, Harvey et al., 2014; Toews & Brelsford, 2012).

Our findings suggest the divergence of these *G. cancriformis* groups likely occurred sometime around the Pliocene/Pleistocene boundary, which coincides with the final uplift of this part of the Andes (Gregory‐Wodzicki, 2000). However, we are unable to pinpoint an exact population divergence date. This is because differences in effective population sizes can also affect time estimates, either overestimating them (with mtDNA) or underestimating them (with nDNA). In addition, the use of standard substitution rates provides precise but inaccurate time estimates (although this effect is reduced when analyzing closely related species or population data) (dos Reis, Donoghue, & Yang, 2015).

In contrast, the Western and Central Colombian Cordilleras, as well as the Dry Diagonal in Brazil, do not seem to contribute to the diversification of these spider populations. This contrasts with previous reports where the Brazilian Dry Diagonal has been found as a natural barrier to gene flow in taxa such as frogs, birds, and lizards (Fouquet, Santana Cassini, Fernando Baptista Haddad, Pech, & Trefaut Rodrigues, 2014; Harvey & Brumfield, 2015; Werneck, Gamble, Colli, Rodrigues, & Sites, 2012). The genetic homogeneity found within the wEC clade may also be explained by the topography of the Western and Central Colombian Cordilleras, which are considerably narrower than the Eastern one (Guarnizo et al., 2015), and which might have facilitated the dispersal of individuals across this region.

Species diversification driven by the uplift of the Andes has been documented in several organisms including birds (Chaves, Pollinger, Smith, & LeBuhn, 2007; Fernandes, Wink, Sardelli, & Aleixo, 2014; Ribas, Moyle, Miyaki, & Cracraft, 2007), reptiles (Teixeira et al., 2016), amphibians (García‐R et al., 2012; Guarnizo, Amézquita, & Bermingham, 2009; Guarnizo et al., 2015), bees (Dick et al., 2004), and butterflies (Elias et al., 2009); however, the persistence of gene flow between populations separated by the Andes is far less known (Hoffmann & Baker, 2003; Miller et al., 2008; Oswald et al., 2017). Here, despite the vicariance associated with the Andean uplift that resulted in eEC and wEC Andean clades for *G. cancriformis*, we found individuals with shared ancestry between the main two geographic groups. The approximate likelihood demographic model implemented, identified gene flow as the most likely explanation for this. Furthermore, the model with the best support implies divergence in the face of gene flow after τ/5 generations forward in time, which suggests a short allopatric period.

Altitudinal depressions across the Andes can contribute to the dispersal of individuals, thus allowing admixture between populations that occur at opposite sides of this barrier. In fact, the EC of the Colombian Andes is not a uniform barrier along its length and has at least two depressions, the Andalucía pass and the Suaza‐Pescado valleys, which may be acting as dispersal corridors (Cadena et al., 2016). To our knowledge, scenarios consistent with Andean altitudinal depressions facilitating dispersion and gene flow have only been reported in the Peruvian Porculla pass, where six codistributed bird taxon pairs showed asynchronous divergence times, likely due to independent dispersal events coupled with gene flow (Oswald et al., 2017). In arthropods, there is evidence for dispersal across the Andes (Dick et al., 2004), but the persistence of gene flow across this barrier has not been demonstrated. We hypothesize that eEC and wEC populations of *G*. *cancriformis* have used such type of passes to cross the Eastern Cordillera and reproduce with populations on opposite sides, even after they achieved some degree of divergence. This could be facilitated by aerial dispersal mechanisms such as ballooning, where the friction between the air and the spider's silk can make an individual move up to 3,200 Km (Gressitt, 1965). Although this displacement strategy has not been observed in *G*. *cancriformis*, it is used by its sister taxa (Bell, Bohan, Shaw, & Weyman, 2005).

Color polymorphism in the opisthosoma of *G. cancriformis* did not explain the structure found in this species. However, the lack of association of mtDNA or nDNA haplotypes with coloration may be due to the nature of the loci studied, as they evolve neutrally and are not members of any known pigmentation pathway in arthropods (Wittkopp & Beldade, 2009). It is also important to bear in mind that information of color phenotype is not available for all samples (mainly for those from Brazil), which may be hindering a phenotype by genotype association. This seems, however, unlikely as most of the samples within the wEC clade have phenotype information and still do not cluster by phenotype. Even so, the mtDNA phylogenetic pattern suggests, to some extent, that this polymorphism pre‐dates the geographic split. Otherwise, the genetic connectivity between the populations at both sides of the Andes may be favoring the flow of color alleles thus maintaining phenotypic variation. This remains to be tested.

The scenario of vicariance coupled with gene flow found here supports the original ideas of Chapman (1917, 1926) and Haffer (1967a,b), who claimed that the similarities in the composition of the flora and fauna at both sides of the Andes might be due to dispersals through altitudinal depressions in the cordilleras (Chapman, 1917, 1926; Haffer, 1967a,b). However, because we are studying populations of a single species, the signatures of allele sharing we obtained could also be the result of incomplete lineage sorting (ILS), especially affecting nuclear loci. By bad luck, determining the extent to which gene flow and ILS contribute to such shared genetic variation is difficult (Holder, Anderson, & Holloway, 2001; Kubatko, 2009; Kubatko & Degnan, 2007). A more exhaustive evaluation of the role of the Andes and gene flow in the diversification of *G. cancriformis* would require the inclusion of more populations at the lowlands and at both sides of the Andes (especially those closer to altitudinal depressions in the cordilleras). This sampling would also allow further investigation into questions such as the origin and maintenance of color polymorphism in this spider.

This case constitutes one of the few phylogeographic studies in arthropods, and the first arachnids, showing that the Andes acts as a permeable barrier rather than an absolute one for the free movement of alleles. However, our findings are based on a limited sample of the genome. Therefore, further work on this topic implementing genomic approaches is needed, especially in invertebrates. This will allow the implementation of comprehensive analyses of comparative phylogeography to understand how geographic barriers and genetic connectivity across them shape genetic diversity and impact genetic structure.

## CONFLICT OF INTEREST

All authors declare no conflict of interest.

## AUTHOR CONTRIBUTIONS

F.C.S‐R., E.L.P., C.S., and V.N.S. conceived the idea and designed the experiments. F.C.S.‐R. collected the individuals. F.C.S.‐R. and C.P‐D. processed the samples in the laboratory and got the sequences. V.N.S. C.S. and C.F.A. contributed with material, tools, and reagents. F.C.S‐R. and C.S. analyzed the data. F.C.S.‐R, C.S., and C.P‐D wrote the manuscript. All authors approved the final version submitted.

## DATA AVAILABILITY

Sequences were deposited in GenBank under accession numbers MH258252‐MH258747.
