# Supplementary material for: Gene flow and Andean uplift shape the diversification of Gasteracantha cancriformis (Araneae: Araneidae) in Northern South America
Source: Ecol Evol. 2018 Jun 25;8(14):7131–42. doi: 10.1002/ece3.4237 (PMC6065347; doi:10.1002/ece3.4237)
Supplement: Supplementary file 1 [file ECE3-8-7131-s001.docx]

**Supplementary table 1. Samples information**. Individuals 1 to 105 are *Gasteracantha cancriformis* while individuals 106 and 107 correspond to *Micrathena vigorsi.*

| **Number** | **Country** | **State** | **Locality** | **Latitude** | **Longitude** | **Altitude (mts)** | **Color** |
| --- | --- | --- | --- | --- | --- | --- | --- |
| 1 | Brazil | Acre | Acre | -9.9817 | -67.8110 | 201 | NA |
| 2 | Brazil | Acre | Acre | -9.9817 | -67.8110 | 201 | NA |
| 3 | Brazil | Acre | Acre | -9.9817 | -67.8110 | 201 | NA |
| 4 | Brazil | Acre | Acre | -9.9817 | -67.8110 | 201 | NA |
| 5 | Brazil | Acre | Acre | -9.9817 | -67.8110 | 201 | NA |
| 6 | Brazil | Acre | Acre | -9.9817 | -67.8110 | 201 | NA |
| 7 | Brazil | Acre | Acre | -9.9817 | -67.8110 | 201 | NA |
| 8 | Brazil | Bahia | Praia do Forte | -12.5254 | -38.0148 | 20 | NA |
| 9 | Brazil | Bahia | Praia do Forte | -12.5254 | -38.0148 | 20 | NA |
| 10 | Brazil | Bahia | Praia do Forte | -12.5254 | -38.0148 | 20 | NA |
| 11 | Brazil | Bahia | Praia do Forte | -12.5254 | -38.0148 | 20 | NA |
| 12 | Brazil | Bahia | Praia do Forte | -12.5254 | -38.0148 | 20 | NA |
| 13 | Brazil | Bahia | Praia do Forte | -12.5254 | -38.0148 | 20 | NA |
| 14 | Brazil | Bahia | Praia do Forte | -12.5254 | -38.0148 | 20 | NA |
| 15 | Brazil | Bahia | Praia do Forte | -12.5254 | -38.0148 | 20 | NA |
| 16 | Brazil | Bahia | Lencois | -12.5605 | -41.3859 | 390 | NA |
| 17 | Brazil | Bahia | Lencois | -12.5605 | -41.3859 | 390 | NA |
| 18 | Brazil | Bahia | Lencois | -12.5605 | -41.3859 | 390 | NA |
| 19 | Brazil | Bahia | Lencois | -12.5605 | -41.3859 | 390 | NA |
| 20 | Brazil | Bahia | Lencois | -12.5605 | -41.3859 | 390 | NA |
| 21 | Brazil | Bahia | Lencois | -12.5605 | -41.3859 | 390 | NA |
| 22 | Brazil | Bahia | Lencois | -12.5605 | -41.3859 | 390 | NA |
| 23 | Brazil | Bahia | Lencois | -12.5605 | -41.3859 | 390 | NA |
| 24 | Brazil | Sao Paulo | Campinas | -22.8189 | -47.0698 | 610 | NA |
| 25 | Colombia | Tolima | Ibagué | 4.4281 | -75.2129 | 1170 | NA |
| 26 | Colombia | Tolima | Ibagué | 4.4281 | -75.2129 | 1170 | NA |
| 27 | Colombia | Tolima | Ibagué | 4.4281 | -75.2129 | 1170 | NA |
| 28 | Colombia | Tolima | Ibagué | 4.4281 | -75.2129 | 1170 | NA |
| 29 | Colombia | Tolima | Ibagué | 4.4281 | -75.2129 | 1170 | NA |
| 30 | Colombia | Tolima | Ibagué | 4.4281 | -75.2129 | 1170 | White |
| 31 | Colombia | Tolima | Ibagué | 4.4444 | -75.1916 | 1070 | White |
| 32 | Colombia | Tolima | Ibagué | 4.4444 | -75.1916 | 1070 | Yellow/Orange |
| 33 | Colombia | Tolima | Ibagué | 4.4444 | -75.1916 | 1070 | White |
| 34 | Colombia | Tolima | Ibagué | 4.4444 | -75.1916 | 1070 | Yellow |
| 35 | Colombia | Tolima | Ibagué | 4.4444 | -75.1916 | 1070 | Yellow |
| 36 | Colombia | Tolima | Armero | 5.0023 | -74.9077 | 280 | Yellow |
| 37 | Colombia | Tolima | Armero | 5.0023 | -74.9077 | 280 | White |
| 38 | Colombia | Tolima | Armero | 5.0023 | -74.9077 | 280 | Yellow |
| 39 | Colombia | Tolima | Armero | 5.0023 | -74.9077 | 280 | Yellow |
| 40 | Colombia | Guajira | Palomino | 11.2516 | -73.5584 | 0 | White |
| 41 | Colombia | Guajira | Palomino | 11.2516 | -73.5584 | 0 | White |
| 42 | Colombia | Guajira | Palomino | 11.2516 | -73.5584 | 0 | White |
| 43 | Colombia | Guajira | Palomino | 11.2516 | -73.5584 | 0 | White |
| 44 | Colombia | Valle del Cauca | Cali | 3.5678 | -76.5739 | 1900 | White |
| 45 | Colombia | Valle del Cauca | Cali | 3.5678 | -76.5739 | 1900 | Orange |
| 46 | Colombia | Valle del Cauca | Cali | 3.5678 | -76.5739 | 1900 | White |
| 47 | Colombia | Valle del Cauca | Cali | 3.5678 | -76.5739 | 1900 | Black |
| 48 | Colombia | Valle del Cauca | Cali | 3.5678 | -76.5739 | 1900 | White |
| 49 | Colombia | Valle del Cauca | Cali | 3.5678 | -76.5739 | 1900 | White |
| 50 | Colombia | Valle del Cauca | Cali | 3.5678 | -76.5739 | 1900 | White |
| 51 | Colombia | Valle del Cauca | Cali | 3.5678 | -76.5739 | 1900 | White |
| 52 | Colombia | Meta | Villavicencio | 4.0727 | -73.5869 | 390 | White_Black |
| 53 | Colombia | Meta | Villavicencio | 4.0727 | -73.5869 | 390 | NA |
| 54 | Colombia | Meta | Villavicencio | 4.0727 | -73.5869 | 390 | White |
| 55 | Colombia | Meta | Villavicencio | 4.0727 | -73.5845 | 398 | White |
| 56 | Colombia | Meta | Villavicencio | 4.0727 | -73.5845 | 398 | White |
| 57 | Colombia | Meta | Villavicencio | 4.1644 | -73.5813 | 396 | White |
| 58 | Colombia | Meta | Villavicencio | 4.1644 | -73.5813 | 396 | White |
| 59 | Colombia | Meta | Villavicencio | 4.1644 | -73.5813 | 396 | Yellow |
| 60 | Colombia | Meta | Villavicencio | 4.1644 | -73.5821 | 396 | Yellow |
| 61 | Colombia | Meta | Villavicencio | 4.1644 | -73.6416 | 486 | White |
| 62 | Colombia | Meta | Villavicencio | 4.0671 | -73.7114 | 502 | White_Black |
| 63 | Colombia | Meta | Villavicencio | 4.0671 | -73.7114 | 502 | White_Black |
| 64 | Colombia | Meta | Villavicencio | 4.0671 | -73.7114 | 502 | White |
| 65 | Colombia | Meta | Villavicencio | 4.0671 | -73.7114 | 502 | White_Black |
| 66 | Colombia | Meta | Villavicencio | 4.0671 | -73.7114 | 502 | White_Black |
| 67 | Colombia | Meta | Villavicencio | 3.6128 | 73.7913 | 424 | White |
| 68 | Colombia | Meta | Villavicencio | 3.6166 | 73.7990 | 398 | Orange |
| 69 | Colombia | Meta | Villavicencio | 3.6128 | 73.7913 | 424 | Yellow |
| 70 | Colombia | Meta | Villavicencio | 3.6128 | 73.7913 | 424 | Orange |
| 71 | Colombia | Meta | Buenavista | 4.1758 | -73.6813 | 1100 | White_Black |
| 72 | Colombia | Meta | Buenavista | 4.1758 | -73.6813 | 1100 | White_Black |
| 73 | Colombia | Meta | Buenavista | 4.1758 | -73.6813 | 1100 | Orange |
| 74 | Colombia | Meta | Buenavista | 4.1758 | -73.6813 | 1100 | Orange |
| 75 | Colombia | Meta | Buenavista | 4.1758 | -73.6813 | 1100 | White_Black |
| 76 | Colombia | Quindio | Boquia | 4.6375 | -75.5872 | 1750 | Orange |
| 77 | Colombia | Quindio | Boquia | 4.6375 | -75.5872 | 1750 | White |
| 78 | Colombia | Quindio | Boquia | 4.6375 | -75.5872 | 1750 | Orange |
| 79 | Colombia | Quindio | Boquia | 4.6375 | -75.5872 | 1750 | Black |
| 80 | Colombia | Quindio | Boquia | 4.6375 | -75.5872 | 1750 | White |
| 81 | Colombia | Quindio | Boquia | 4.6375 | -75.5872 | 1750 | Yellow |
| 82 | Colombia | Quindio | Boquia | 4.6375 | -75.5872 | 1750 | Yellow |
| 83 | Colombia | Quindio | Boquia | 4.6375 | -75.5872 | 1750 | White |
| 84 | Colombia | Valle del Cauca | Bahia Malaga | 3.9833 | -77.3500 | 32 | White |
| 85 | Colombia | Valle del Cauca | Bahia Malaga | 3.9833 | -77.3500 | 32 | White |
| 86 | Colombia | Valle del Cauca | Bahia Malaga | 4.1023 | -77.4912 | 7 | Yellow |
| 87 | Colombia | Valle del Cauca | Bahia Malaga | 4.1023 | -77.4912 | 7 | White |
| 88 | Colombia | Valle del Cauca | Bahia Malaga | 4.1023 | -77.4912 | 7 | Yellow |
| 89 | Colombia | Valle del Cauca | Bahia Malaga | 4.1023 | -77.4912 | 7 | Orange |
| 90 | Colombia | Valle del Cauca | Bahia Malaga | 4.1023 | -77.4912 | 7 | Orange |
| 91 | Colombia | Valle del Cauca | Bahia Malaga | 4.1023 | -77.4912 | 7 | Orange |
| 92 | Colombia | Valle del Cauca | Bahia Malaga | 4.1023 | -77.4912 | 7 | NA |
| 93 | Colombia | Amazonas | Leticia | -4.1813 | -69.9520 | 76 | NA |
| 94 | Colombia | Santander | Bucaramanga | 7.1426 | -73.1196 | 970 | Orange |
| 95 | Colombia | Santander | Bucaramanga | 7.1426 | -73.1196 | 970 | White |
| 96 | Colombia | Santander | Bucaramanga | 7.1426 | -73.1196 | 970 | NA |
| 97 | Colombia | Valle del Cauca | Palmira | 4.1786 | -76.2058 | 940 | White |
| 98 | Colombia | Valle del Cauca | Palmira | 4.1786 | -76.2058 | 940 | Yellow |
| 99 | Colombia | Antioquia | Medellin | 6.2079 | -75.5699 | 1530 | Orange |
| 100 | Colombia | Antioquia | Medellin | 6.2079 | -75.5699 | 1530 | White |
| 101 | Colombia | Choco | Cocalito | 6.3853 | -77.3999 | 80 | Orange |
| 102 | Colombia | Sucre | Tolu | 9.5932 | -75.5710 | 5 | White |
| 103 | Colombia | Sucre | Tolu | 9.5932 | -75.5710 | 5 | White |
| 104 | Colombia | Sucre | Tolu | 9.5932 | -75.5710 | 5 | Orange |
| 105 | Colombia | Sucre | Tolu | 9.5932 | -75.5710 | 5 | White |
| 106 | Colombia | Amazonas | Leticia | -4.1813 | -69.9520 | 76 | NA |
| 107 | Colombia | Amazonas | Leticia | -4.1813 | -69.9520 | 76 | NA |

**Supplementary table 2. Output of G-PhoCS. θ** = population genetic diversity; τ= divergence time scalar; *m*= migration rate; eEC=east of the eastern Colombian cordillera; wEC=west of the eastern Colombian cordillera.

| **Gamma priors (α, β)** | **Loci** | | **𝞱_ eEC** | **𝞱_ wEC** | **𝞱_root** | **𝞃_root** | **m_eEC → wEC** | **m_wEC → eEC** |
| --- | --- | --- | --- | --- | --- | --- | --- | --- |
| α=1, β=30 | mtDNA | mean | 6.79E-03 | 1.01E-02 | 1.36E-02 | 1.88E-02 | 0.1038 | 9.81E-02 |
|  |  | 95% HPD upper | 1.03E-02 | 1.50E-02 | 2.07E-02 | 2.83E-02 | 0.2445 | 0.2313 |
|  |  | 95% HPD lower | 3.64E-03 | 5.47E-03 | 7.39E-03 | 7.36E-03 | 4.41E-03 | 4.16E-03 |
|  |  | ESS | 9721.546 | 11895.21 | 24406.05 | 1.39E+03 | 691.6436 | 691.6437 |
|  | nDNA | mean | 4.26E-03 | 2.80E-02 | 4.69E-03 | 5.54E-04 | 9.94E-02 | 0.1054 |
|  |  | 95% HPD upper | 7.07E-03 | 6.21E-02 | 7.46E-03 | 8.10E-04 | 0.2317 | 0.2456 |
|  |  | 95% HPD lower | 1.89E-03 | 7.30E-03 | 2.14E-03 | 3.10E-04 | 1.54E-03 | 1.63E-03 |
|  |  | ESS | 3176.885 | 2405.156 | 7332.42 | 2565.706 | 561.9101 | 561.9104 |
| α=1, β=300 | mtDNA | mean | 6.64E-03 | 1.08E-02 | 6.26E-03 | 1.55E-02 | 0.102 | 9.86E-02 |
|  |  | 95% HPD upper | 1.04E-02 | 1.57E-02 | 1.51E-02 | 2.46E-02 | 0.2451 | 0.236 |
|  |  | 95% HPD lower | 3.27E-03 | 6.44E-03 | 1.20E-03 | 4.48E-03 | 3.42E-03 | 3.30E-03 |
|  |  | ESS | 26026.37 | 20626.13 | 1.53E+03 | 7.05E+02 | 479.4454 | 479.4453 |
|  | nDNA | mean | 4.23E-03 | 1.34E-02 | 4.46E-03 | 5.44E-04 | 1.03E-01 | 0.102 |
|  |  | 95% HPD upper | 6.80E-03 | 2.16E-02 | 7.12E-03 | 7.90E-04 | 0.2451 | 0.2422 |
|  |  | 95% HPD lower | 1.93E-03 | 6.42E-03 | 2.06E-03 | 3.00E-04 | 2.57E-03 | 8.54E-03 |
|  |  | ESS | 4209.072 | 6113.141 | 7546.608 | 2725.821 | 421.1546 | 421.1544 |

**Supplementary table 3. Results of the Analysis of Molecular Variance by loci.** Columns are coded as follows: df: degrees of freedom; ss: sum of squares; vc: variance components; vp: variation percentages; fi: fixation index.

| **Mitochondrial** | | | | | |  |
| --- | --- | --- | --- | --- | --- | --- |
| **Source of variation** | **df** | **ss** | **vc** | **vp** | **fi** | **p-value** |
| Among regions | 1 | 584 | 10 | 58.04 | F_CT_=0.58039 | < 0.001 |
| Among populations within regions | 16 | 359 | 3 | 18.68 | F_ST_=0.76718 | < 0.001 |
| Within populations | 87 | 366 | 4 | 23.28 | F_SC_=0.44516 | < 0.001 |
| **ITS** | | | | | |  |
| **Source of variation** | **df** | **ss** | **vc** | **vp** | **fi** | **p-value** |
| Among regions | 1 | 34 | 0.3317 | 24.3 | F_CT_=0.24302 | < 0.001 |
| Among populations within regions | 16 | 14 | 0 | -1.46 | F_ST_=0.22847 | < 0.001 |
| Within populations | 180 | 193 | 11 | 77.15 | F_SC_=0.24302 | < 0.001 |
| **28S** | | | | | |  |
| **Source of variation** | **df** | **ss** | **vc** | **vp** | **fi** | **p-value** |
| Among regions | 1 | 24 | 0.22081 | 24.45 | F_CT_=0.24452 | < 0.001 |
| Among populations within regions | 15 | 10 | 0.00164 | 0.18 | F_ST_=0.24633 | < 0.001 |
| Within populations | 195 | 133 | 0.68061 | 75.37 | F_SC_=0.00241 | < 0.001 |
| **HSP90** | | | | | |  |
| **Source of variation** | **df** | **ss** | **vc** | **vp** | **fi** | **p-value** |
| Among regions | 1 | 22 | 0.18918 | 15.18 | F_CT_=0.15175 | 0.02 |
| Among populations within regions | 14 | 50 | 0.25653 | 20.58 | F_ST_=0.35753 | < 0.001 |
| Within populations | 168 | 134.55 | 0.80089 | 64.25 | F_SC_=0.24260 | < 0.001 |

**Supplementary table 4.** Analysis of isolation by distance for each locus evaluated. (a) Mantel and partial Mantel correlation test (b) Linear regression and correlation coefficients for genetic distances vs. geographic distances.

| **Locus** | **Mantel r** | **p-value** | **Partial Mantel r** | **p-value** |
| --- | --- | --- | --- | --- |
| COI-16S | 0.1014 | 0.2091 | -0.081 | 0.644 |
| ITS | 0.3491 | 0.0061 | 0.1695 | 0.133 |
| 28s | 0.1188 | 0.1699 | -0.1098 | 0.765 |
| Hsp90 | 0.0698 | 0.4433 | 0.0559 | 0.456 |

(b)

| **Locus** | **R** | **R^2^** | **p-value** |
| --- | --- | --- | --- |
| COI-16S | 0.1934 | 0.0324 | 0.0065 |
| ITS | 0.3464 | 0.1099 | 0 |
| 28S | 0.2662 | 0.067 | < 0.001 |
| Hsp90 | 0.1159 | 0.0095 | 0.063 |

**Supplementary table 5.** Measures of goodness of fit of alternative models.

| **Model** | **AIC** | **lnL** | **K** | **∆AIC** | **wAIC** |
| --- | --- | --- | --- | --- | --- |
| 1 | 114.20 | -56.10 | 1 | 15.66 | 1.50E-04 |
| 2 | 101.65 | -48.82 | 2 | 3.10 | 0.08 |
| 3 | 102.26 | -49.13 | 2 | 3.72 | 0.06 |
| 4 | 99.70 | -47.85 | 2 | 1.16 | 0.21 |
| 5 | 101.72 | -47.86 | 3 | 3.18 | 0.08 |
| 6 | 101.78 | -47.89 | 3 | 3.24 | 0.07 |

**Supplementary table 6.** Chi-squared Monte Carlo test for coloration, geographical group separated by the Colombian EC and locality.

| **Locus** | **p-value color** | **p-value eEC-wEC** | **p-value locality** |
| --- | --- | --- | --- |
| Mitocondrial | 0.6317 | < 0.001* | < 0.001* |
| ITS | 0.3261 | < 0.001* | 0.037* |
| 28S | 0.345 | < 0.001* | < 0.001* |
| HSP90 | 0.365 | 0.116 | 0.604 |
