# Supplementary material for: Gene flow and Andean uplift shape the diversification of Gasteracantha cancriformis (Araneae: Araneidae) in Northern South America
Source: Ecol Evol. 2018 Jun 25;8(14):7131–42. doi: 10.1002/ece3.4237 (PMC6065347; doi:10.1002/ece3.4237)
Supplement: Supplementary file 2 [file ECE3-8-7131-s002.docx]

**Supplementary Figure 1.** Divergence time estimates from BEAST (mtDNA) and G-PhoCS (mtDNA and nDNA). Mean values (dots) lower 95% HPD and upper 95% HPD are shown.

**Supplementary Figure 2.** Haplotype median joining networks. Each tick on branches represents a mutational step.

**Supplementary Figure 3.** Heat map of pairwise F_ST_ values between populations from eastern (green bar) and western (red bar) sides of the eastern Colombian cordillera. (a) mitochondrial loci, (b) ITS, (c) 28S and (d) HSP90. Populations (x and y axes) are coded as follows: (a) Acre, (b) Praia do forte, (c) Lencois, (d) Villavicencio, (e) Buenavista, (f) Ibague, (g) Palomino, (h) Cali, (i) Boquia, (j) Bahia Malaga, (k) Bucaramanga, (l) Palmira, (m) Tolu.

**
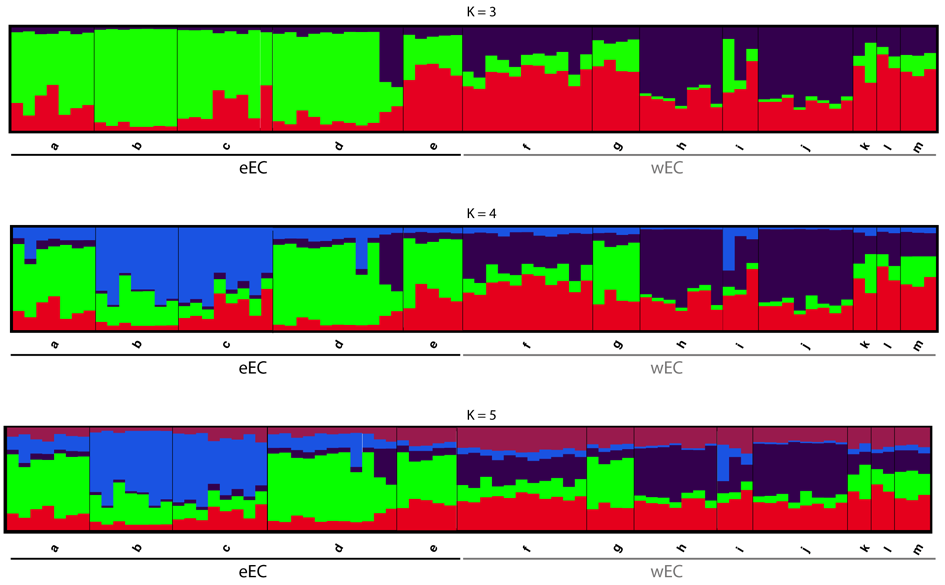
**

**Supplementary figure 4.** Bayesian population assignment test based on the nuclear loci for K=3 (top), K=4 (middle) and K=5 (bottom). Sampled localities (x axis) are: (a) Acre, (b) Praia do forte, (c) Lencois, (d) Villavicencio, (e) Buenavista, (f) Ibague, (g) Palomino, (h) Cali, (i) Boquia, (j) Bahia Malaga, (k) Bucaramanga, (l) Palmira, (m) Tolu. Bars on the bottom indicate the geographical region each population belongs to (east or west of the Eastern Colombian Cordillera).

**Supplementary figure 5.** Optimal K inferred using a) the Evanno method and b) the natural logarithm of the probability of the data.

**Supplementary figure 6.** Canonical discriminant analysis performed with (a) Mitochondrial loci, (b) ITS, (c) 28S and d) HSP90. eEC: east of the eastern Colombian cordillera; wEC: west of the eastern Colombian cordillera.

**Supplementary figure 7.** Phylogenetic tree inferred with mitochondrial data. Support values are depicted in the nodes: posterior probability (top) and bootstrap (bottom). Tips labels correspond to samples reported in Table S1.

**Supplementary figure 8.** Isolation by distance plots by loci. (a) Mitochondrial loci, (b) ITS, (c) 28S and (d) HSP90.


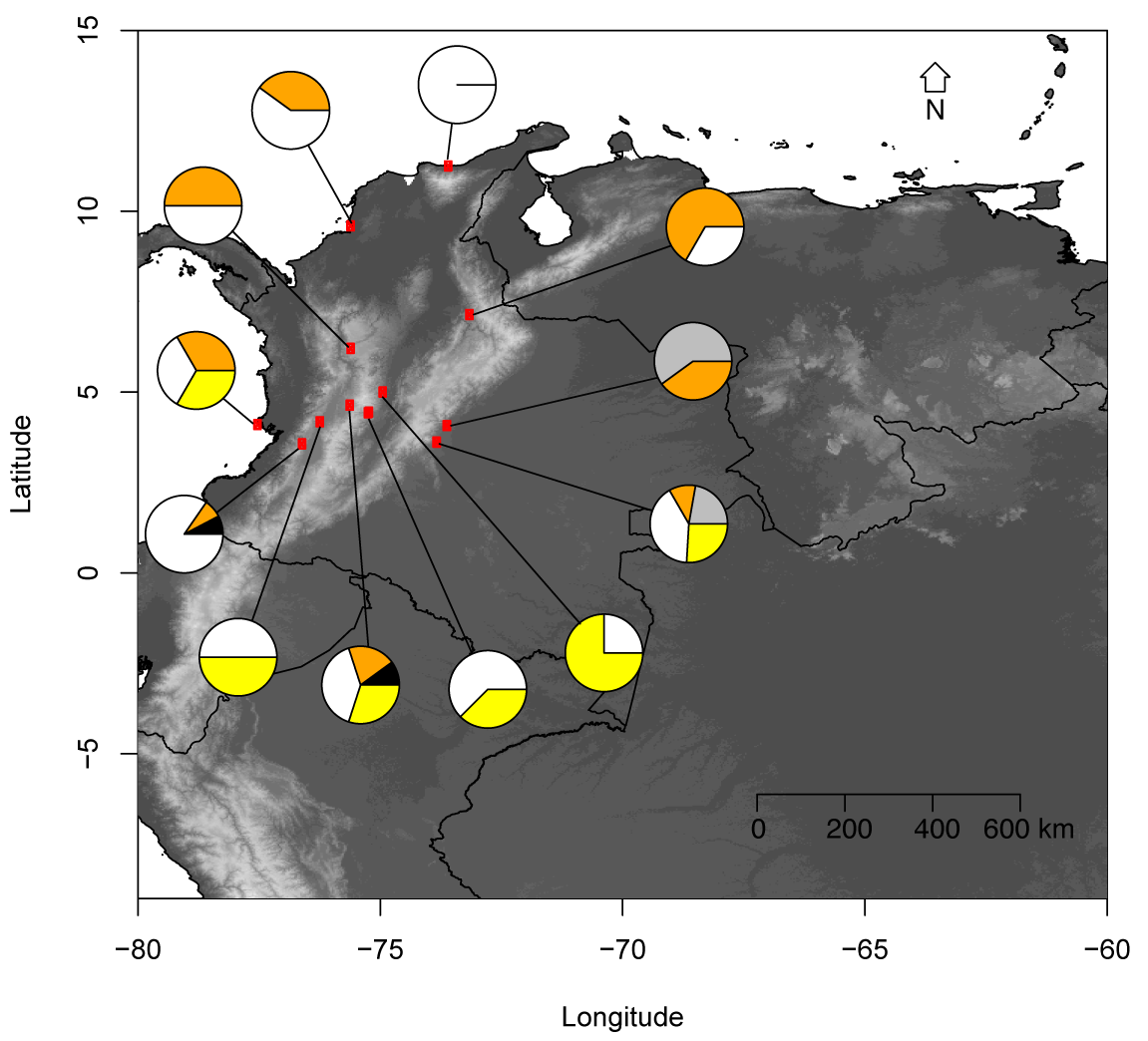


**Supplementary figure 9.** Relative frequencies of color morphs collected in Colombia. The color morphs are those represented in the main text.
